# A CLOSER LOOK AT EMOTIONAL ACCEPTANCE IN LATE LIFE

**DOI:** 10.1093/geroni/igae098.1480

**Published:** 2024-12-31

**Authors:** Claudia Haase, Yu Li, Chen-Wei Yu, Lillian Fu, David Rompilla

**Affiliations:** Northwestern University, Evanston, Illinois, United States; Northwestern University, Evanston, Illinois, United States; Northwestern University, Evanston, Illinois, United States; Northwestern University, Evanston, Illinois, United States; Texas A&M University, College Station, Texas, United States

## Abstract

When older adults regulate emotions, they tend to prefer emotional acceptance (i.e., embracing emotions without judging them) over other strategies, such as reappraisal (i.e., reframing an emotional situation to view it in a more positive light) or detachment (i.e., mental distancing from an emotional situation). While regulation strategies, such as reappraisal, have been widely studied, interest in emotional acceptance is just starting to take off. Here, we present findings from two studies on emotional acceptance with older adults. In a laboratory-based study, we examined habitual emotional regulation (using questionnaires) and instructed emotion regulation (instructing participants to use different emotion regulation strategies when watching loss-themed film clips vs. “just watch” them while monitoring their autonomic physiology) in a sample of 129 older adults (age 64-83). We examined emotional acceptance and (to determine specificity) detachment and reappraisal. Findings showed that habitual emotional acceptance (but not detachment or reappraisal) was associated with (1) greater vagal reactivity during instructed emotion regulation and (2) lower levels of anxiety and depressive symptoms. In an online study, we probed a novel measure of emotional acceptance in a sample of 241 older adults (age 65-88). A confirmatory factor analysis supported a 2-factor solution with acceptance of negative emotions and acceptance of positive emotions as two distinct factors. Together, these findings highlight the physical and mental health benefits of emotional acceptance in late life, underscore differences between different cognitive change strategies, and suggest further probing of accepting not just negative but also positive emotions in future research.

